# An Evolutionary Comparison of the Handicap Principle and Hybrid Equilibrium Theories of Signaling

**DOI:** 10.1371/journal.pone.0137271

**Published:** 2015-09-08

**Authors:** Patrick Kane, Kevin J. S. Zollman

**Affiliations:** 1 Department of Social and Decision Sciences, Carnegie Mellon University, Pittsburgh, PA, United States of America; 2 Department of Philosophy, Carnegie Mellon University, Pittsburgh, PA, United States of America; Universidad de Granada, SPAIN

## Abstract

The handicap principle has come under significant challenge both from empirical studies and from theoretical work. As a result, a number of alternative explanations for honest signaling have been proposed. This paper compares the evolutionary plausibility of one such alternative, the “hybrid equilibrium,” to the handicap principle. We utilize computer simulations to compare these two theories as they are instantiated in Maynard Smith’s Sir Philip Sidney game. We conclude that, when both types of communication are possible, evolution is unlikely to lead to handicap signaling and is far more likely to result in the partially honest signaling predicted by hybrid equilibrium theory.

## Introduction

Signaling is ubiquitous in the biological world. Signaling interactions are usually divided into one of two types: those which involve common interest—where honest communication is in the interest of all parties—and those that involve at least some conflict of interest—where in some cases there is an incentive to lie [1]. Many cases of honest signaling, despite the presence of partial conflict of interest, have presented an evolutionary mystery. Why, if there is an incentive to deceive, is honesty present? Why hasn’t evolution led to dishonesty?

The paradigmatic answer is found in Zahavi’s [2, 3] handicap principle [1, 4–6]. This principle suggests that in order for communication to occur in the presence of conflict of interest, there must be some significant cost to the means of communication—the “signal.” Maynard Smith presented the Sir Philip Sidney game [7] as a concrete illustration of this proposal. In this game a child can communicate its state of need via a single signal. Maynard Smith showed in this model that in order for totally honest communication to be stable, the signal must have a relatively high cost for the child.

In one respect this model, and others like it, underwrite the handicap principle because they show how signal costs can stabilize communication. They nonetheless have a number of shortcomings. First these models only demonstrate the theoretical, rather than empirical plausibility of the handicap principle. This is especially troubling because some empirical studies have failed to find the significant signal cost postulated by the handicap mechanism [6, 8–15] (in contrast, some studies have found high signal cost, for example [16]).

Second, these models only demonstrate that the handicap principle is a possible explanation; they do not demonstrate that signal cost is necessary for the stability of honesty. Indeed, utilizing different models, a number of scholars have demonstrated that there are other potential solutions that can stabilize honest signaling. These all turn on various mechanisms for introducing “marginal” cost—cost imposed on dishonest signaling [17–23].

Third, models of the handicap principle focus entirely on considerations of stability, not evolvability [24]. Even when the signal is sufficiently costly to stabilize honesty in the Sir Philip Sidney game, there remains another important evolutionary outcome—a pooling equilibrium where no information is communicated. It has been shown that in some cases these equilibria are better for both parties than the handicap signaling equilibria [25–28] and that in some contexts they are more likely the result of evolution by natural selection [27].

Finally, these models all focus on the conditions necessary to sustain perfectly honest communication. However, there may be outcomes of evolution where some information is communicated, even if there remains some deception in equilibrium. Three such possibilities have been proposed, partial pooling equilibria [29, 30], “cheating” equilibria [31], and hybrid equilibria [27, 28, 32]. These solutions, along with the marginal costs mechanisms, are all consistent with the low signal costs observed in some experimental tests of the handicap principle. Here we will focus on one of these mechanisms, the hybrid equilibrium. This equilibrium is most closely related to the handicap principle because it exists in many of the traditional models of handicap signaling when the signal cost is too low to sustain completely honest communication.

In all of the various models of signaling, a single model is constructed wherein the particular mechanism is demonstrated. Each model, therefore, adds another mechanism to the list of potential mechanisms without providing a theoretical basis for regarding any mechanism as more plausible than another. Because of this limitation, these models cannot effectively compare the different mechanisms. We here attempt to partially remedy this situation by constructing a model where the handicap, hybrid, and pooling equilibria all co-exist. This will allow us to provide a theoretical comparison of these mechanisms.

Toward this end, we develop a version of Maynard Smith’s Sir Philip Sidney game which has, as equilibria, handicap signaling, pooling, and hybrid equilibria. This allows for a direct comparison of these three equilibria in order to understand what types of signaling should be expected to evolve in the realistic situation where more than one signal type are available. Ultimately we conclude that in reasonable situations where all are possible, we should expect to see the hybrid equilibrium far more often than either the traditional handicap signaling equilibrium or the non-communicative pooling equilibrium. This suggests that in natural populations, partially honest communication ought to be found more often than handicap signaling. From the outset, we should note that this does not rule out alternative explanations mentioned above. It does however, suggest that the handicap principle is less plausible than at least one alternative mechanism for the evolution of communication.

## Sir Philip Sidney game

The Sir Philip Sidney game was devised by Maynard Smith as a simplification of Grafen’s model of signaling [33], applied to the particular case of interaction between offspring and parent. In the Sir Phillip Sidney game there are two players, a parent and a child. The child can be in one of two states: needy or healthy. While the child can directly observe these states the parent cannot, the parent must rely on the child to communicate whether it is needy or healthy. The child does this by either sending a signal and paying some associated cost or remaining silent (which is costless). The parent can then choose whether or not to transfer a resource to the child. The resource is beneficial to whoever receives it. But, the relative benefit is larger to the needy child than it is to the healthy one.

The Sir Philip Sidney game has become almost synonymous with the handicap principle; it is regularly used as one the central illustration of the mechanism [1, 5]. It provides a relatively simple model of signaling interactions between relatives, and it is a plausible models of the handicap principle. For this reason, it represents the best-case scenario for the handicap principle.

To begin, “nature” flips a (biased) coin to decide if the child is healthy or needy. The child is needy with probability *p* healthy with probability 1 − *p*. The child observes its state of need and then sends the costly signal or refrains from signaling. Upon observing whether the child signaled or not, the parent chooses to either transfer the resource or not. Should the child receive the resource its fitness is 1 regardless of whether it was needy or healthy. However, if a needy child does not receive the resource, its fitness is 1 − *a*. A healthy child who does not get the resource has a fitness of 1 − *b* (where *a* > *b* > 0). In addition, if the child sends the costly signal, then the child pays a cost *c* > 0.

If the parent keeps the resource it receives a payoff of 1. If the parent transfers the resource it receives a payoff of 1 − *d* (where 1 > *d* > 0). Finally, because we assume that the parent and child are related to some degree, there is a relatedness parameter *k*.

Some equilibria of this game feature no communication between child and parent because child never sends the signal. These are called “pooling equilibria” because all types are pooled into the cost-free signal. In these equilibria the parent cannot differentiate between healthy and needy children. (See [25, 27] for the conditions necessary to sustain various pooling equilibria.)

Central to the handicap principle are the handicap-signaling equilibria, where the signals carry meaning and affect the actions of the parent. In order for there to be a signaling equilibrium, the parent must prefer to transfer the resource to the needy child but not the healthy one, i.e. $a\geq \frac{d}{k}\geq b$. The literature usually focuses on situations of partial conflict of interest, where the parent prefers to keep the resource when the child is healthy, but where the child wants to manipulate the parent into providing it. This conflict of interest arises when *b* > *kd*.

Handicap-signaling is only possible when the needy child is willing to pay the cost in order to secure the resource, but the healthy child is unwilling. This requires that *a* ≥ *c* + *kd* ≥ *b*. This equation represents the apparent mathematical confirmation of the handicap principle. When there is conflict of interest, there must be a significant cost in order to sustain honest communication in equilibrium.

While these conditions establish the stability of signaling, they do not establish that handicap signaling is likely to evolve over the alternative pooling equilibria which are present for all parameter values. In order to consider this possibility we must explicitly model the process of evolution in games of this sort.

Following the analysis of the Sir Philip Sidney game in [27] we use the two-population replicator dynamics [34, 35]. The two population replicator dynamics consists of two sets of differential equations,

$${\dot{x}}_{i}=x_{i}\left(\pi \left(i,y\right)-\pi \left(x,y\right)\right)$$

$${\dot{y}}_{j}=y_{j}\left(\pi \left(j,x\right)-\pi \left(y,x\right)\right)$$

Where *x*_*i*_ represents the frequency of type *i* in the child population, and *y*_*j*_ represent the frequency of type *j* in the parent population. The types are child and parent strategies, respectively, in the Sir Philip Sidney game (listed in Table 1). *π*(*i*, **y**) represents the expected fitness of child strategy *i* against the parent population, represented by **y** (similarly for the parent strategies against the child population). *π*(**x**, **y**) represents the average fitness of all types in the child population

**Table 1:** Sender and donor strategies in the Sir Philip Sidney Game.

	Child strategies		Parent strategies
*S*_1_:	Signal if healthy	*R*_1_:	Transfer if no signal
*S*_2_:	Signal if needy	*R*_2_:	Transfer if signal
*S*_3_:	Never Signal	*R*_3_:	Never transfer
*S*_4_:	Always Signal	*R*_4_:	Always transfer

It is shown in [27] that both the signaling and pooling equilibria are stable in the two-population replicator dynamics. In addition, they show that for certain values of the parameters the pooling equilibria is a more likely result of evolution via natural selection than the handicap signaling equilibrium.

In order to generalize these results, we consider 1,000 random instances of the Sir Philip Sidney game where handicap signaling is an equilibrium. We choose values for *a*, *b*, *c*, *d*, *k*, and *p* uniformly at random from the interval [0, 1] for each parameter, but satisfying the constraints listed above. For each individual game, we choose 1,000 random frequencies for each population and consider the evolution of the system according to the two population replicator dynamics. (We utilize Euler’s method, where we update for 10,000 generations. This is equivalent to using the discrete time two-population replicator dynamics, for a discussion see [34, 36].)

**Fig 1:**
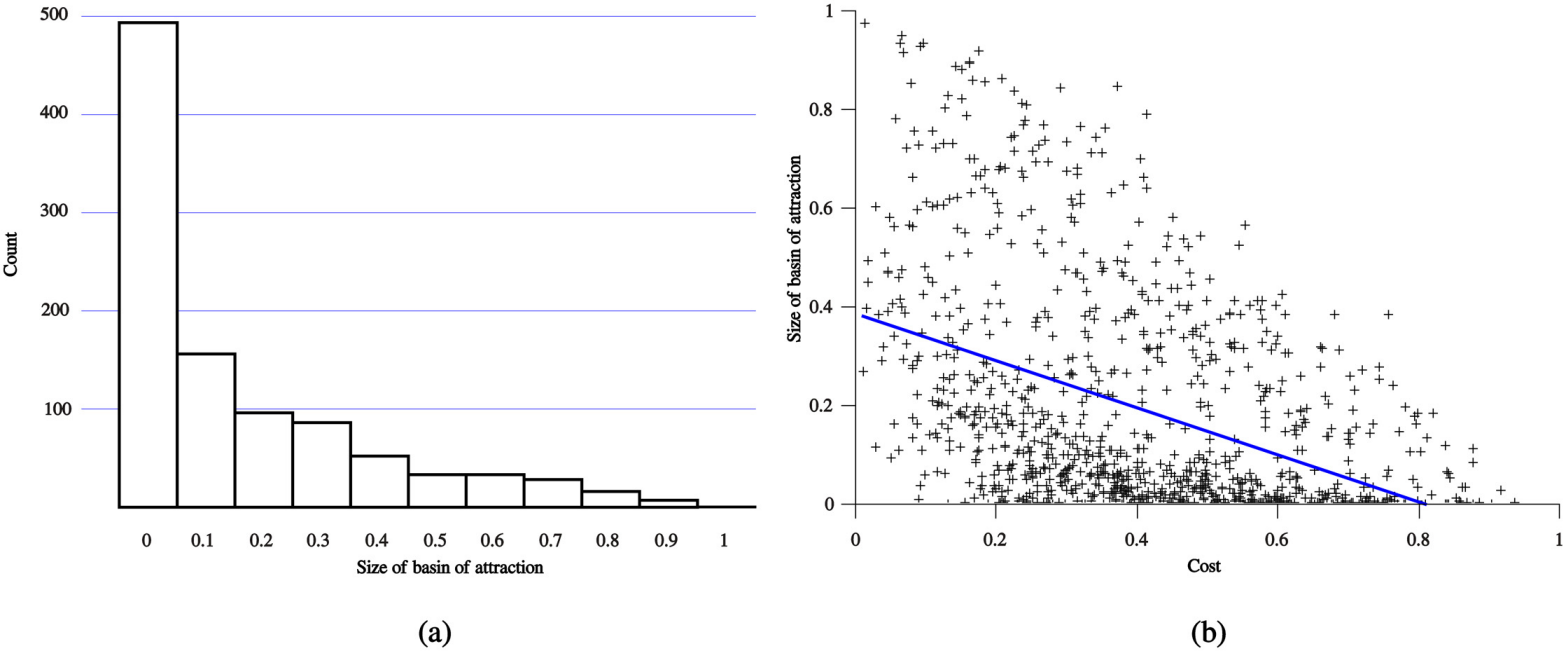
Size of basin of attraction for handicap signaling. Simulation results from a random sample of instances of the Sir Philip Sidney game where the parameters satisfy the conditions necessary for conflict of interest and the existence of the signaling equilibrium. (a) shows the probability that a randomly chosen initial population evolves to the signaling equilibrium. (b) illustrates the relationship between the probability of evolving signaling and the underlying cost of the signal.

Fig 1(a) shows the probability that handicap signaling will evolve in a randomly chosen instance of the Sir Philip Sidney game. These simulations reveal that signaling is relatively unlikely to evolve when the cost is sufficiently high to maintain honest communication. Almost half of the simulations resulted in an extremely small basin of attraction for handicap-signaling.

While far from perfect, the cost of the signal appears strongly correlated with the probability of evolving to a signaling equilibrium, as illustrated in Fig 1B. Lower signal costs increase the probability of evolving to a handicap-signaling equilibrium.

Following work by Wagner [32], Huttegger and Zollman [27] show that when there is conflict of interest, but when the signal is insufficiently costly, there exists another type of equilibrium called a *hybrid equilibrium*. In this equilibrium the child randomizes between two strategies: *signal only when needy* and *always signal*. The parent also randomizes between two strategies: *transfer only if signal is observed* and *never transfer*.

In this equilibrium *some* information is communicated, but not as much as in the signaling equilibrium. Wagner [32] and Zollman, Bergstrom, and Huttegger [28] show that this equilibrium is not unique to the Sir Philip Sidney game, but is a generic property of a number of different signaling games that feature partial conflict of interest.

The hybrid equilibrium bears some similarity to partial pooling equilibria [29, 30] and “cheating” equilibria [31]. In partial pooling equilibria partially honest communication is achieved by “pooling” together several different types. Originally proposed in [29], these were shown to exist in aversion of the Sir Philip Sidney game with more than two states of need [30]. These differ from the hybrid equilibrium in that they involve monomorphic populations where two different types always send the same signal. In the hybrid equilibrium, there is a polymorphic population with deceptive and non-deceptive types. In this respect, the hybrid equilibrium is most similar to the “cheating” equilibria discussed in [31]. Cheating equilibria were demonstrated to exist in games modeling animal contests, and feature a mixture of honest and dishonest signaling.

In the Sir Philip Sidney game, there are three conditions necessary for the existence of the hybrid equilibrium. The first is the condition which ensures the parent wishes to tranfser to the needy child but not to the healthy one, $a\geq \frac{d}{k}\geq b$. The second is that signal costs are too low to sustain the signaling equilibrium, *b* > *c* + *kd*. And finally, it must be the case that, in ignorance of the state of the child, the parent prefers to withhold the resource, *d* > *k*(*ma* + (1 − *m*)*b*).

When these conditions are satisfied the two parent and two child strategies combine to create a game similar to the well known game matching pennies. The parent’s best response to *signal only when needy* is to transfer the resource to a signaling child. If the parent will transfer to a signaling child, then the child’s best response to adopt the strategy *always signal* (because *b* > *c* + *kd*). If the child adopts this strategy, then the parent’s best response is to never transfer (because *d* > *k*(*ma* + (1 − *m*)*b*)). And finally, when the parent never transfers, the cost of the signal ensures that the child would prefer to never signal.

As in matching pennies, when there exists a best-response cycle of this type, there is a unique, mixed strategy Nash equilibrium. Huttegger and Zollman [27] illustrate this remains an equilibrium when the other four strategies are introduced.

**Fig 2:**
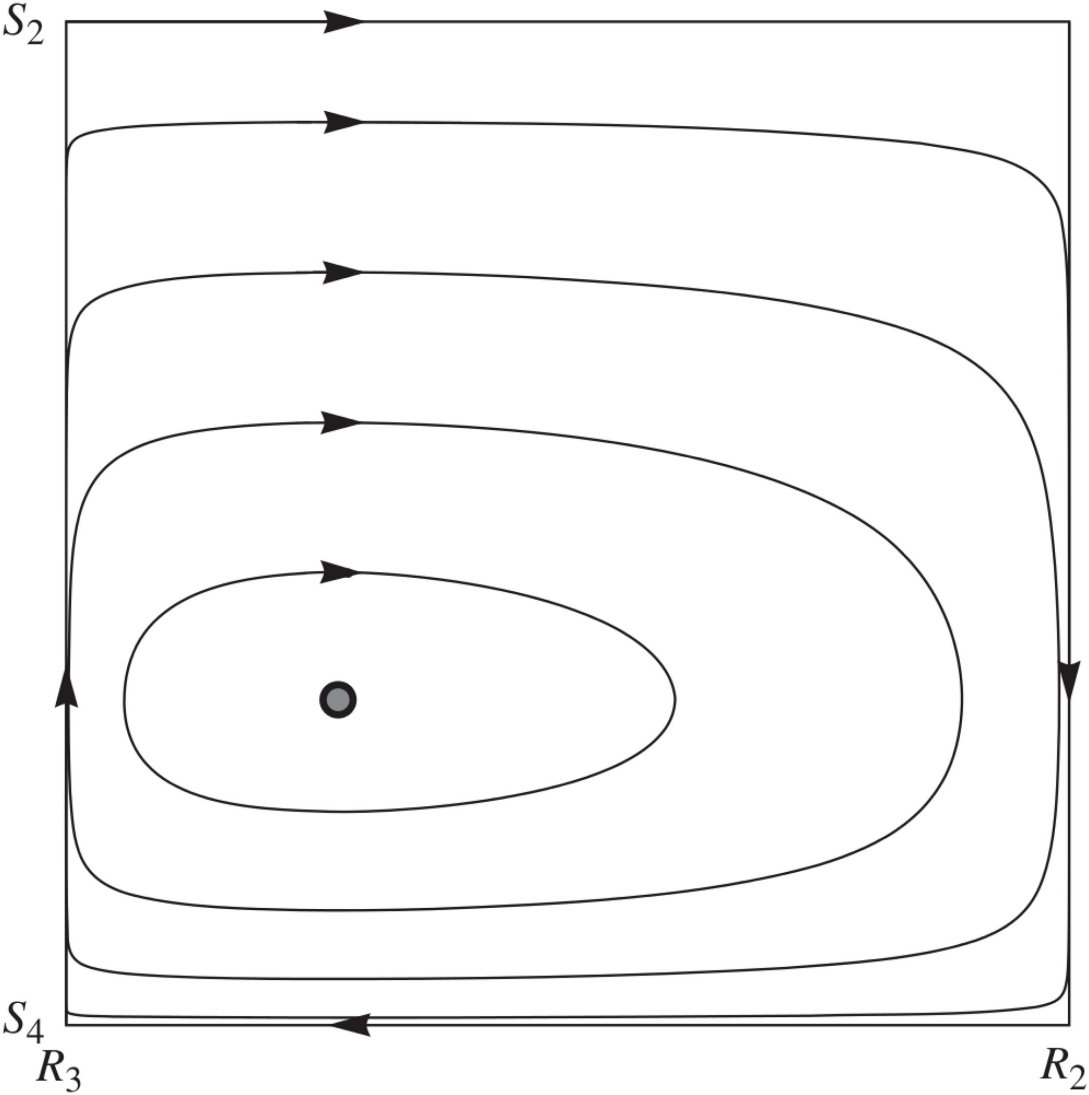
Phase portrait of hybrid equilibrium. From [27]. This illustrates the phase portrait of the two population replicator dynamics on the plane comprised by the four hybrid equilibrium strategies. The child strategies are *signal only when needy* (*S*_2_) and *always signal* (*S*_4_), and the parent strategies are *transfer only if the signal is observed* (*R*_2_) and *never transfer* (*R*_3_).

This equilibrium exists on a plane comprised by the two child strategies and the two parent strategies. Consider an arbitrary child population where only the two hybrid equilibrium child-strategies are present paired with an arbitrary parent population with only the parent’s hybrid-equilibrium strategies. Any pair of populations on this plane will cycle around the hybrid equilibrium in a closed orbit, illustrated by Fig 2. Including all parent and child strategies, any pair of populations that are near the plane containing the hybrid equilibrium will evolve toward the plane. This illustrates that the hybrid equilibrium represents a potentially plausible end-point of evolution [27].

It does not, however, indicate how likely such a state is to evolve. Because pooling equilibria remain, it is possible that the hybrid equilibrium is only accessible from a relatively small number of initial states that are already relatively close to the equilibrium. Huttegger and Zollman [27] show that for particular parameter values the hybrid equilibrium can evolve from a relatively large number of initial states. Furthermore, the hybrid equilibrium is more likely to evolve than is the signaling equilibrium for a corresponding set of parameter values.

While Huttegger and Zollman considered only a particular assignment of values to the parameters, we can generalize their method and consider a set of random parameterizations of the Sir Philip Sidney game to determine how likely it is that the hybrid equilibrium will emerge in an arbitrary context. As before, we choose 1,000 values for all parameters uniformly at random but satisfying the constraints for the existence of the hybrid equilibrium. Because evolution near the hybrid equilibrium is much slower, we allowed the individual populations to evolve for 50,000 generations before checking their final state.

**Fig 3:**
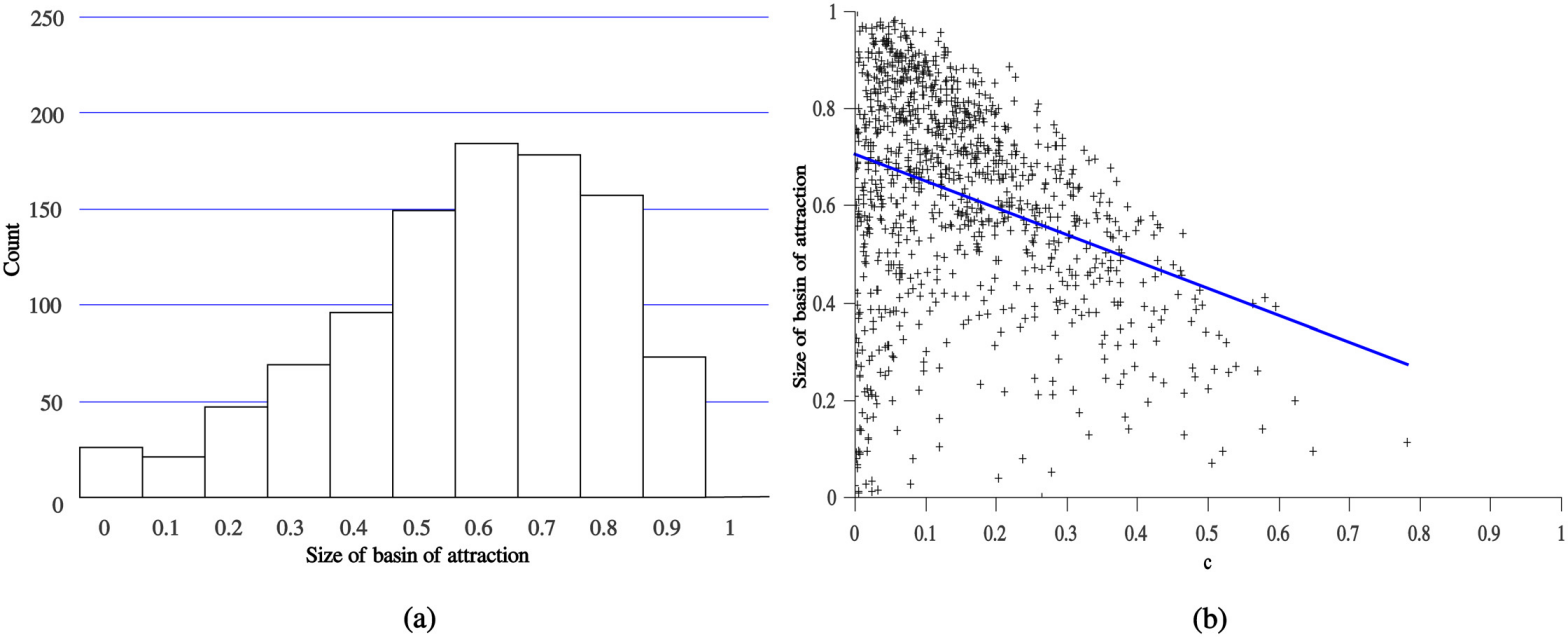
Size of basin of attraction for hybrid equilibrium. Simulation results for a random sample of instances of the Sir Philip Sidney game where the parameters satisfy conflict of interest and the conditions necessary for the existence of the hybrid equilibrium. (a) shows the probability that a randomly chosen initial population evolves to the hybrid equilibrium. (b) illustrates the relationship between the probability of evolving to the hybrid equilibrium and the underlying cost of the signal.

Fig 3A shows that the hybrid equilibrium is significantly more likely to evolve in a randomly chosen Sir Philip Sidney game than the signaling equilibrium is to evolve in the corresponding random sample of games. A similar relationship exists between the probability of evolving this type of communication and the signal cost (see Fig 3B). Importantly, the hybrid equilibrium appears most likely when the cost is relatively low, but not extremely low. As the costs approach zero, the location of the hybrid equilibrium moves closer to the location of the pooling equilibrium where the parent never transfers. Apparently, if they become too close it becomes difficult to evolve an effective system of partial information transfer.

## Multiple signals

The results reported in the previous section are suggestive. When signal costs are lower, some communication is likely to evolve. A population playing a game with a low cost signal is more likely to evolve to a partially communicative equilibrium (the hybrid equilibrium) than is a different population playing a game with a high cost signal is to evolve to a perfectly communicative equilibrium.

This makes the following conclusion tempting: if a single population had access to multiple signals with different costs that it would be more likely to evolve to the hybrid equilibrium than the perfectly communicative equilibrium. But this is an extrapolation from limited evidence. In this section we present a version of the Sir Philip Sidney game with both a high and low cost signal in order to determine which equilibrium is more likely to evolve when both are present.

The underlying game stays very close to the original Sir Philip Sidney game. There are two states of need, healthy and needy. The child is in one of these two states. The parent has a resource that can be transferred or withheld. The only modification is that there are now three strategies available to the child. She can send a high-cost signal and pay cost *c*_*h*_, send a low cost signal and pay *c*_*l*_, or refrain from signaling and pay no cost. In order to preserve the co-existence of both signaling and hybrid equilibria, we will require that *c*_*h*_ > *b* − *kd* > *c*_*l*_ > 0. This condition entails that the traditional signaling equilibrium exists and utilizes *c*_*h*_ while *c*_*l*_ is never used. The hybrid equilibrium also exists and utilizes *c*_*l*_ and where *c*_*h*_ is never used.

The addition of a second signal requires that the organism has access to at least two phenotypes which could be used for signaling. Given the prevalence of “multi-modal” signaling [37–39], where many different phenotypes are used for communication simultaneously, this seems a plausible assumption. In addition, we are assuming some structure to the costs for the two signals. First, one signal must be sufficiently costly to sustain handicap-signaling. Because we are interested in studying a best-case scenario for the handicap principle, we include this possibility here. Second, the other signal must be of lower cost. Empirical studies have regularly found low cost signals [6, 8–15].

The equilibrium structure of this game is very similar to the original Sir Philip Sidney game. There are pooling equilibria where the child refrains from sending either signal and the parent transfers or withholds the resource (depending on the underlying probabilities of the child’s state) regardless of the signal sent.

There is a perfectly communicative equilibrum where the needy child sends the expensive signal and the healthy child refrains from signaling and where the parent transfer the resource only if the expensive signal is sent.

**Fig 4:**
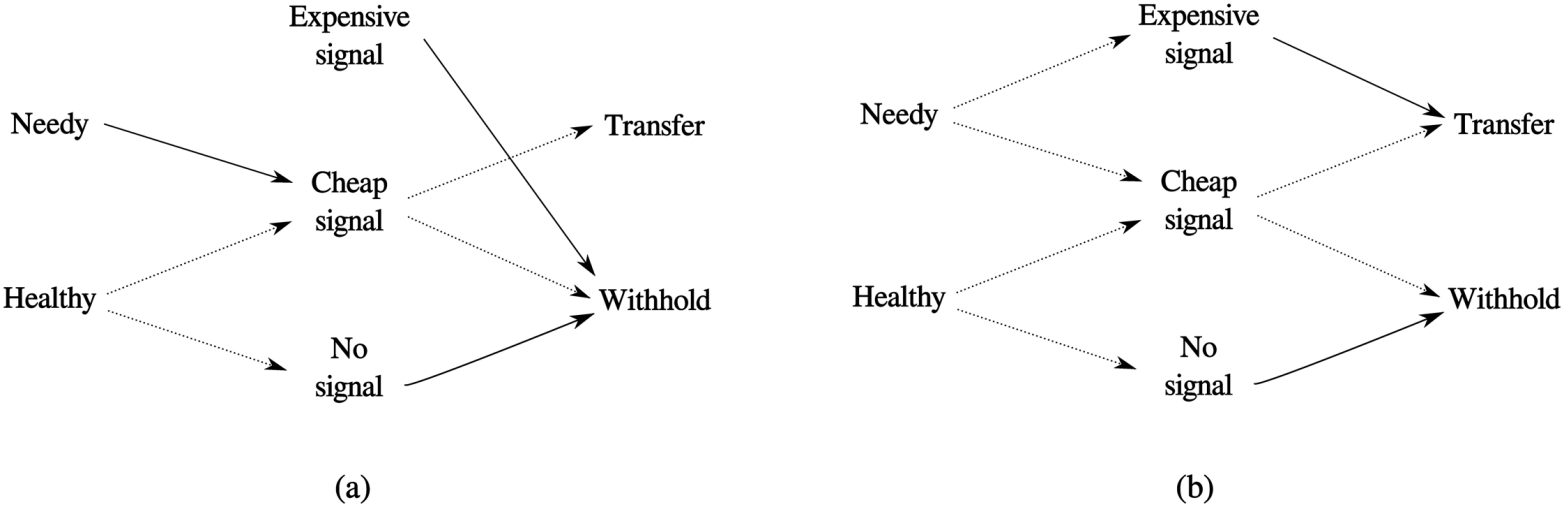
Illustration of the two hybrid equilibria in the Sir Philip Sidney game with two signals. Solid lines represent play with probability 1, dotted lines represent play with non-unity, positive probabilities. The lines on the left represent the strategies of the child, while lines on the right illustrate the strategy of the parent. (a) represents a version of the hybrid equilibria most closely related to the one found in [27]. (b) represents a new hybrid equilibrium which is discussed in more detail in the Appendix.

There are two hybrid equilibria which exist in this game as well. The one pictured in Fig 4A closely resembles the hybrid equilibrium discussed by Huttegger and Zollman. The one pictured in Fig 4B is new with the invention of this game. It only exists for certain parameter values, and is of limited evolutionary significance, the details are presented in the Appendix.

**Fig 5:**
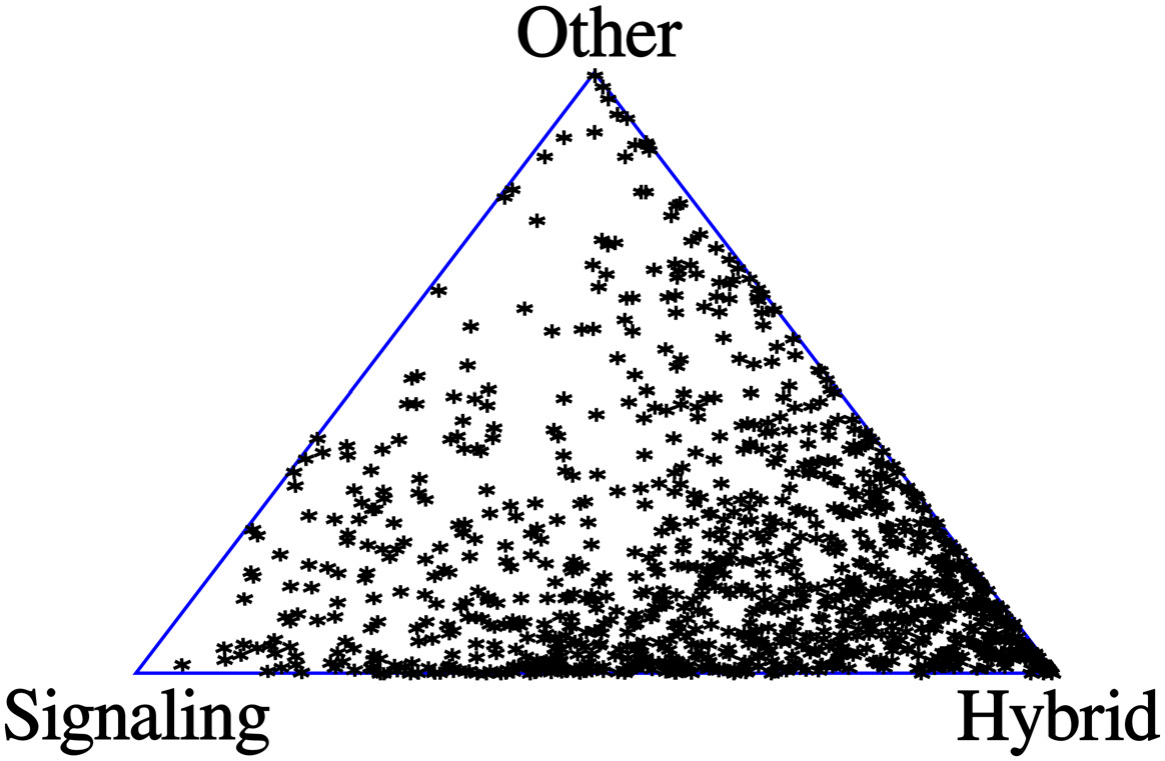
A simplex indicating the probability of evolving to the handicap-signaling equilibrium, hybrid equilibrium, or some other outcome in a randomly chosen Sir Philip Sidney game. Each point represents one random setting of the parameter values. Its location in the simplex indicates the size of the basin of attraction for the various outcomes. The size of the basin of attraction is inversely proportional to its distance from the vertex. For example, if the point is close to the lower right vertex, this indicates that the basin of attraction for the hybrid equilibrium is significantly larger than for the other two outcomes.

To provide the most direct comparison possible, we consider a randomly chosen game with both a separating and a hybrid equilibrium. As before, we generated 1,000 random values for *a*, *b*, *c*_*l*_, *c*_*h*_, *d*, *k*, and *p* such that a signaling equilibrium exists which utilizes *c*_*h*_ and a hybrid equilibrium exists that uses *c*_*l*_. For each parameter value, we then estimate the size of the basin of attraction for each of two equilibria: the hybrid equilibrium utilizing *c*_*l*_ and the signaling equilibrium utilizing *c*_*h*_. We allowed the system to evolve for 50,000 generations before checking the final state. Fig 5 provides a graphical representation of these results. One can see immediately that for a vast majority of instantiations of the Sir Philip Sidney game, the hybrid equilibrium is a more likely outcome of evolution than the traditional signaling equilibrium.

**Fig 6:**
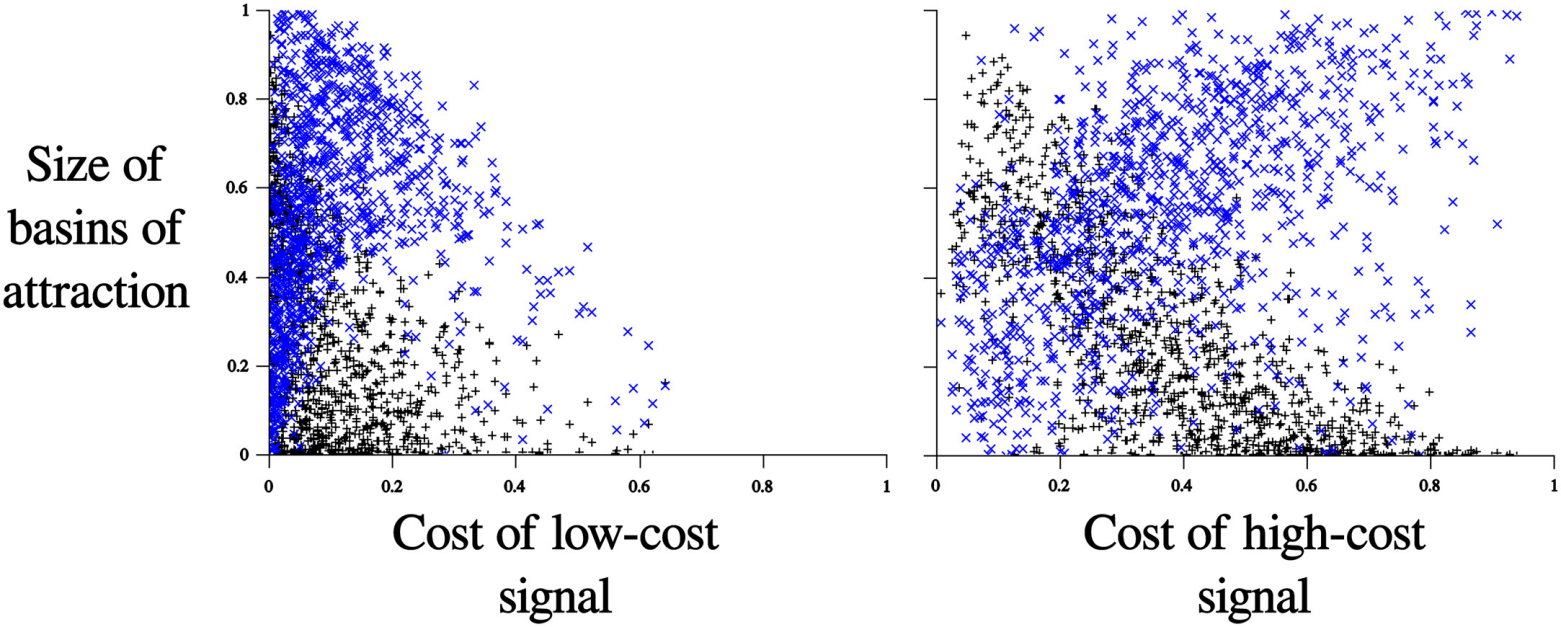
The relationship between the size of the basins of attraction and the two cost parameters. Blue x’s represent the size of the basin of attraction of the hybrid equilibrium, black crosses represent the size of the basin of attraction of the handicap-signaling equilibrium. The plot on the left displays the relationship between the size of the basins of attraction and the cost of the low cost signal, *c*_*l*_. The plot on the right shows the relationship between the size of the basins of attraction and the cost of the high cost signal, *c*_*h*_.

Again we can compare this for various costs, which is illustrated in Fig 6. From this diagram we can infer that in order for handicap-signaling to be more likely than the hybrid equilibria, it must be the case that the high cost signal has very low cost (a best-case scenario for handicap-signaling) and that the low cost signal must also have very low cost (a worst-case scenario for the hybrid equilibrium).

## Discussion

Traditional discussions of the handicap principle have focused on proving the plausibility of the handicap mechanism for maintaining the stability of signaling. However, rather less attention has been paid the evolutionary plausibility of the handicap theory. Through a more exhaustive search of the parameter space, we first illustrated the difficulty of evolving handicap signals. Unless the signal’s cost is very close to the lowest allowable cost, it can be very difficult for a population to evolve a system of communication utilizing that signal.

Because the Sir Philip Sidney game was constructed for the purpose of providing an illustration of the handicap principle, it represents a “best-case” scenario for testing the handicap principle. Even in these favorable circumstances, the handicap mechanism is difficult to evolve; this is telling. Furthermore, that the hybrid equilibrium is more likely to evolve in almost identical conditions suggests that we should not expect to often find handicap signaling in the wild. This explains why some empirical observation have not found substantial signal costs [6, 8–15].

Much of the analysis of the evolution of handicap-signaling, including this one, has utilized the two-population replicator dynamics. This models the parent population and child population as evolving independently. There are many similarities between the dynamics of the two-population replicator dynamics and the one-population replicator dynamics applied to the symmetrized game [36]. But, the similarities are not perfect, and future work should investigate this in the, less tractable, single population dynamics.

Theoretical and empirical problems with the handicap principle have led to an interest in alternatives [23]. We show that one of these alternatives, the hybrid equilibrium, does not suffer from some of the theoretical problems that plague handicap-signaling. The hybrid equilibrium can be relatively easy to evolve in a wide range of signal costs. In addition, the hybrid equilibrium is far more likely to evolve than the handicap-signaling equilibrium in context where both are possible. It is only when the signal cost is almost zero that the hybrid equilibrium represents an implausible evolutionary outcome.

We compare only one alternative—the hybrid equilibrium—to the handicap theory. Thus one should not conclude from this study that the hybrid equilibrium is the most plausible mechanism for signaling. Further work should be conducted comparing the other alternatives to the handicap mechanism and to each other.

As with previous work, this analysis is restricted to a single evolutionary dynamics which is deterministic and features no mutation. Huttegger and Zollman [40] show that, in a game similar to the Sir Philip Sidney game, the significance of the hybrid equilibrium increases when mutation is introduced. Further work is needed to determine whether the equilibrium remains significant when stochastic factors, like drift, are modeled explicitly.

Hybrid equilibria have been shown to exist in a number of games with structures similar to the Sir Philip Sidney game [28, 32]. So far all models of the hybrid equilibrium consider games where the space of relevant states (in this case whether the child is needy or healthy), the space of possible signals, and the space of possible responses is discrete. (These have come to be called “discrete action–response games” [17, 19].) There exist other signaling models where some or all of these are continuous—for example, where the child’s state of need is a variable drawn from an interval [25, 30, 33, 41].

It is unclear the extent to which nature is better modeled by the discrete or continuous games. In many biological contexts, continuous variables become discrete because of the existence of important thresholds or discrete responses (mating is perhaps the most clear example of such a case). But, undoubtedly some biological interactions more closely resemble the continuous model than the discrete one. If a hybrid-like equilibrium were to exist in these continuous games it would look somewhat different because it would likely require that the child adopt a probability distribution over all possible signals for each type (and similarly for the parent). Little is known about the existence of these equilibria in these contexts, and future work should endeavor to see if they exist.

Honesty in communication systems where there is an incentive for deception provides a vexing evolutionary mystery. While the handicap principle provides one potential solution for this mystery, the results reported here suggest it is a rather implausible one. Instead, the hybrid equilibrium explanation offers a more plausible alternative.

Further work is needed to determine if the hybrid equilibrium exists in nature. Zollman, Bergstrom, and Huttegger [28] note that in some cases, this equilibrium may look like a noisy version of the signaling equilibrium unless the proper statistical tests are performed. As a result, the same data might be used, with novel statistical tests, to look for the hybrid equilibrium. They suggest a number of salient features of the hybrid equilibrium which would lend themselves to empirical test. These include variation in both the child population with respect to signaling behavior and variation in the parent population in responsiveness to the signal. This current study suggests that the hybrid equilibrium is more plausible than the handicap mechanism, and is therefore worthy of empirical study.

## Appendix

The edition of a second costly signal creates the possibility of another kind of hybrid equilibrium beyond those observed in [27]—pictured in Fig 4(b). This equilibrium consists of the child randomizing between the expensive and cheap signals when needy and randomizing between the cheap signal and no signal when healthy while the parent always transfers when given the expensive signal, never transfers when given the no signal and randomizes between donating and not donating when they receive the cheap signal.

The parent is randomizing over two strategies: transfer only if sent the expensive signal and transfer if sent either the cheap or expensive signal. We begin by assuming that the child sends either the expensive signal or the cheap signal when needy (with probabilities 1 − *m* and *m* respectively) and sends the cheap signal or no signal when healthy (with probabilities *n* and 1 − *n* respectively). If the parent receives the expensive signal or no signal they have perfect information and act accordingly (by donating and not donating respectively). If the parent receives the cheap signal, however the parent only has partial information about the state of the child. The probability, *x*, that the child is needy given that the cheap signal was sent is given by:

$$x=\frac{mp}{mp+n(1-p)}$$

In order for the new hybrid equilibrium to occur it must be the case that the parent is indifferent between donating and not donating when they receive the cheap signal (additionally it must be the case that the parent wants to transfer given the expensive signal and does not given no signal in other words *a* ≥ *d*/*k* ≥ *b*). Thus the following equality must hold:

$$k\left(1-c_{l}\right)+1-d=xk\left(1-a-c_{l}\right)+\left(1-x\right)k\left(1-b-c_{l}\right)+1$$

where the left side represents donating and the right side represents not donating. This equation can be simplified to:

$$x=\frac{d-kb}{k(a-b)}$$

which must fall between zero and one since *x* is a probability.

When we turn to the child’s side we discover that this equilibrium only exists in very particular circumstances. Suppose that the parent transfers with probability *q* upon receipt of the low cost signal. Because the child mixes between sending the cheap signal when healthy and not sending a signal when healthy it must be the case that the healthy child is indifferent between sending and not sending the signal. This requirement entails the following constraint on *q*:

$$q=\frac{c_{l}}{b-kd}$$

But, it also must be the case that the needy child is indifferent between sending the expensive and the cheap signal. This imposes this constraint on *q*:

$$q=1+\frac{c_{l}-c_{h}}{a-kd}$$

These two equations impose a constraint on the parameters of the Sir Philip Sidney game. They are jointly satisfiable, but they reduce the parameter space by one-dimension. As such if we were to generate instances of the Sir Philip Sidney game at random, those games that make this equilibrium possible would have probability zero. Because of this, this new hybrid equilibrium does not represent a plausible evolutionary outcome.

## Supporting Information

S1 File Code for simulations of the original Sir Philip Sidney game. This file contains the code for simulations of the original Sir Philip Sidney game used to generate the data in paper. Written in NetLogo,
(NLOGO)

S2 File Code for simulations of the three signal Sir Philip Sidney game. This file contains the code for simulations of the three signal Sir Philip Sidney game used to generate the data in paper. Written in NetLogo.
(NLOGO)

S3 File This file contains the data used to generate Fig 1. (QTI)

S4 File This file contains the data used to generate Fig 3. (QTI)

S5 File This file contains the data used to generate Figs 5 and 6. (QTI)
